# SARS-CoV-2 infection and recurrence of anti-glomerular basement disease: a case report

**DOI:** 10.1186/s12882-021-02275-4

**Published:** 2021-02-27

**Authors:** Alexander Winkler, Emanuel Zitt, Hannelore Sprenger-Mähr, Afschin Soleiman, Manfred Cejna, Karl Lhotta

**Affiliations:** 1 Department of Internal Medicine 3 (Nephrology and Dialysis), Feldkirch Academic Teaching Hospital, Carinagasse 47, A-6800 Feldkirch, Austria; 2 Pathology, Cytodiagnostics and Molecular Pathology, Hall in Tirol, Austria; 3 Institute for Diagnostic and Interventional Radiology, Feldkirch Academic Teaching Hospital, Feldkirch, Austria

**Keywords:** Anti-GBM disease, SARS-CoV-2, COVID-19, Glomerulonephritis, Case report

## Abstract

**Background:**

Anti-glomerular basement membrane disease (GBM) disease is a rare autoimmune disease causing rapidly progressive glomerulonephritis and pulmonary haemorrhage. Recently, an association between COVID-19 and anti-glomerular basement membrane (anti-GBM) disease has been proposed. We report on a patient with recurrence of anti-GBM disease after SARS-CoV-2 infection.

**Case presentation:**

The 31-year-old woman had a past medical history of anti-GBM disease, first diagnosed 11 years ago, and a first relapse 5 years ago. She was admitted with severe dyspnoea, haemoptysis, pulmonary infiltrates and acute on chronic kidney injury. A SARS-CoV-2 PCR was positive with a high cycle threshold. Anti-GBM autoantibodies were undetectable. A kidney biopsy revealed necrotising crescentic glomerulonephritis with linear deposits of IgG, IgM and C3 along the glomerular basement membrane, confirming a recurrence of anti-GBM disease. She was treated with steroids, plasma exchange and two doses of rituximab. Pulmonary disease resolved, but the patient remained dialysis-dependent. We propose that pulmonary involvement of COVID-19 caused exposure of alveolar basement membranes leading to the production of high avidity autoantibodies by long-lived plasma cells, resulting in severe pulmonary renal syndrome.

**Conclusion:**

Our case supports the assumption of a possible association between COVID-19 and anti-GBM disease.

## Background

Anti-glomerular basement disease (anti-GBM disease) is a rare small-vessel vasculitis. Characterized by the presence of circulating antibodies directed against the non-collagen NC1 domain of the alpha3 chain of collagen type IV in glomerular and alveolar basement membranes, the disease manifests as rapidly progressive crescentic glomerulonephritis and, in 40 to 60% of cases, with pulmonary haemorrhage [1].

Recently, a report from London described a five-fold increased incidence of anti-GBM disease during the coronavirus pandemic. Four of the eight reported cases tested positive for SARS-Cov-2 IgM antibodies [2]. We here report the case of a young woman who experienced a recurrence of anti-GBM disease following an infection with the new coronavirus.

## Case presentation

The now 31-year-old Caucasian woman, a heavy smoker (17 pack-years), had her first attack of anti-GBM disease in 2009 with life-threatening pulmonary haemorrhage and rapidly progressive glomerulonephritis. Plasma anti-GBM antibodies were detected (titre 137 U/ml), and a renal biopsy showed linear IgG deposits along the glomerular basement membrane. She was treated with 1 g iv prednisolone for 3 days followed by 80 mg orally, plasma exchange, and six cycles of iv cyclophosphamide 750 mg. After an allergic reaction to fresh frozen plasma, therapy was switched from plasma exchange to immunoadsorption using Ig-Therasorb® columns with Sepharose-coupled polyclonal sheep antibodies against human immunoglobulins. She responded clinically well to treatment and anti-GBM antibodies became negative. Her serum creatinine in remission was 168 μmol/L. Eight months later she acquired an influenza H1N1 virus infection with pneumonia and adult respiratory distress syndrome requiring mechanical ventilation. In 2012, parvovirus B19-associated perimyocarditis resulting in dilated cardiomyopathy was diagnosed. She was treated with intravenous immunoglobulin. A second episode of parvovirus B19-positive perimyocarditis occurred in 2015. Two months after the infection, the patient was re-admitted with haemoptysis, deteriorating renal function and a nephritic urinary sediment. A test for anti-GBM antibodies was borderline (titre 20 U/mL). She was treated with 500 mg iv prednisolone for 3 days followed by 80 mg orally, plasma exchange and two 1-g doses of rituximab. Serum creatinine stabilized in the range of 180 μmol/L.

In September 2020, the patient presented critically ill with severe dyspnoea, haemoptysis and anaemia. Heart rate was 120/min, blood pressure 170/100 mmHg and oxygen saturation 93% when breathing ambient air. Within 1 week haemoglobin had fallen from 9.6 to 7.1 g/dL. Serum creatinine increased from around 270 μmol/L to 420 μmol/L. Urinalysis revealed 3+ proteinuria, 4+ haematuria and a highly nephritic sediment. Table 1 shows the most relevant results of laboratory investigations at first presentation of the patient.

**Table 1:** Main laboratory results on admission

Parameter	Result	Reference Range
Haemoglobin	9.6 g/dL	12–16 g/dL
Thrombocytes	247 G/L	150–450 G/L
Leukocytes	11.0 G/L	3.4–10.4 G/L
Lymphocytes	1.3 G/L	0.7–4.8 G/L
D-dimer	0.95 mg/dL	< 0.50 mg/dL
C-reactive protein	2.94 mg/dL	< 0.50 mg/dL
Procalcitonin	0.25 ng/mL	< 0.50 ng/mL
Creatinine	420 μmol/L	44.5–71.2 μmol/L
Urinary protein-creatinine ratio	3.2 g/g	< 0.11 g/g

### Radiology studies of the patient are presented in Fig. 1

A SARS-CoV-2 RT-PCR was positive with a cycle threshold (Ct) > 30. SARS-CoV-2 antibodies were undetectable by ELISA. A diagnosis of COVID-19 was made, and the patient was treated with conventional oxygen supplementation and dexamethasone 6 mg daily. Because of the medical history, haemoptysis and renal disease a recurrence of anti-GBM disease was suspected. Tests for anti-GBM antibodies and anti-neutrophil cytoplasmic antibodies were negative. A renal biopsy was performed. Of seven glomeruli three were completely sclerosed and three revealed fibrocellular crescents with florid segmental fibroid necrosis (Fig. 2a). Focal tubular atrophy with low-grade lymphomononuclear infiltration and moderate interstitial fibrosis were found. Immunohistochemistry revealed linear deposits of IgG, IgM and C3 along the glomerular basement membrane (Fig. 2b). No staining on tubular basement membranes was observed.

**Fig. 1:**
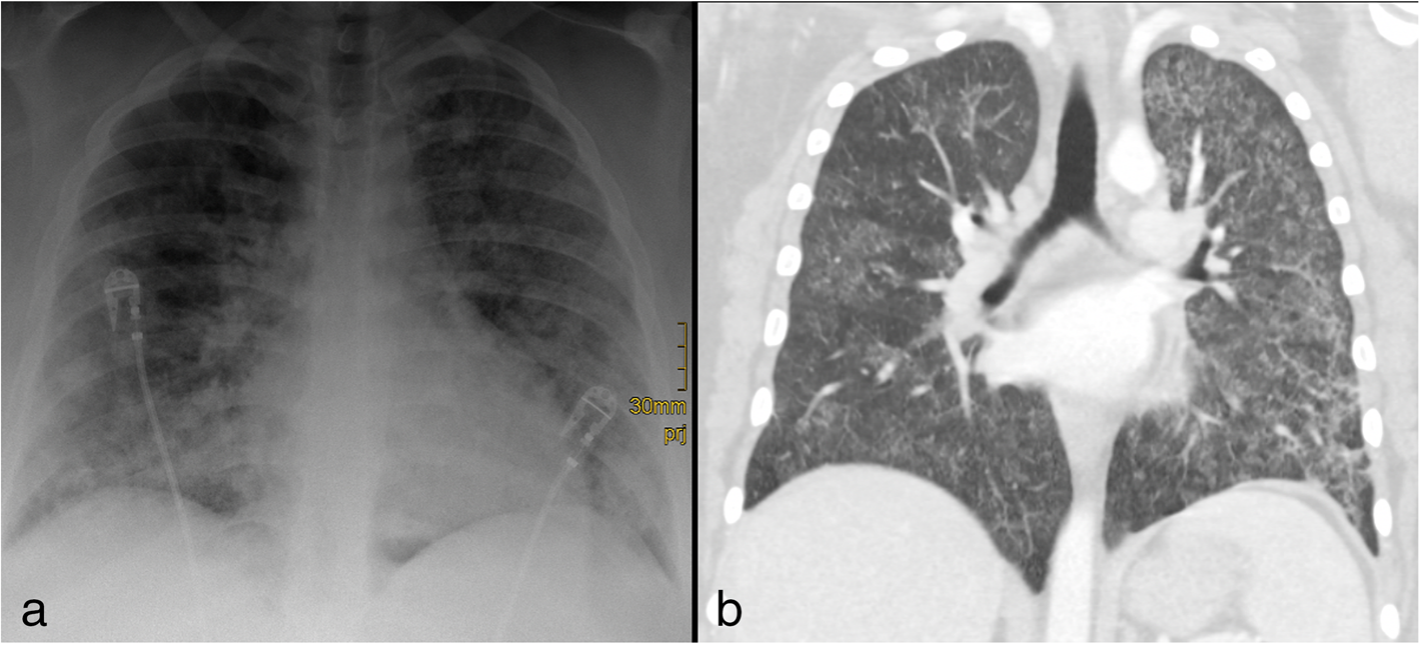
**a** Chest x-ray at admission showed diffuse ground glass opacifications with minimal peripheral consolidations. **b** Four days later a CT scan of the chest revealed diffuse bilateral ground-glass opacities with minimal peripheral consolidations

**Fig. 2:**
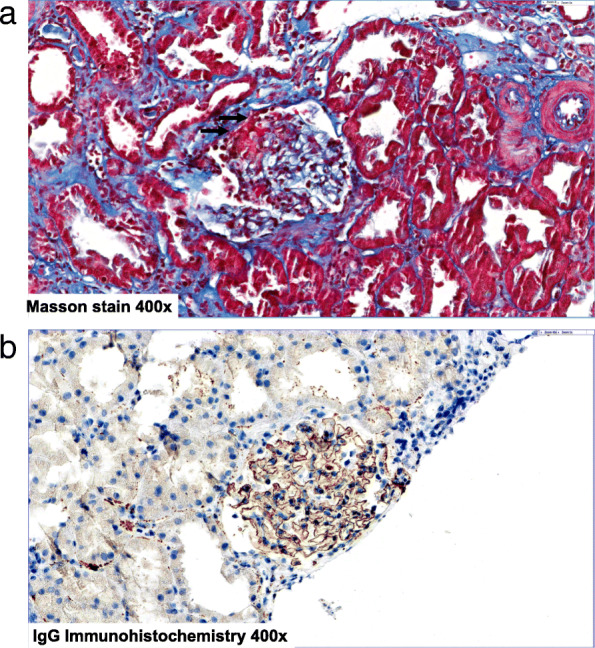
**a** glomerular capillary necrosis and acute extravasation of fibrin into Bowman’s space (arrows). **b** Linear deposits of IgG on the GBM

The diagnosis of recurrent anti-GBM disease was made. The patient was switched from dexamethasone to 60 mg prednisolone, which was reduced to 40 mg after 2 weeks and tapered to 10 mg over the next 2 months. Furthermore, she received one dose of 1 g iv cyclophosphamide and two 1-g doses of rituximab. Cotrimoxazole was given for *pneumocystis jirovecii* prophylaxis. A series of seven plasma exchanges of four litres each led to remission of haemoptysis, and her clinical condition improved. After a symptom-free interval of 1 week, haemoptysis re-occurred. Another series of seven plasma exchanges was performed followed by rapid remission of pulmonary symptoms. The patient, however, remained dialysis-dependent. A  time line of diagnoses and treatment modalities is provided in Table 2.

**Table 2:** Time line of diagnoses and treatments

Date	Diagnosis	04/2009	12/2009	09/2012	03/2015	05/2015	08/2020?	09/2020
		Anti-GBM disease	H1N1 pneumonia	Parvovirus B19 Myocarditis	Parvovirus B19 Myocarditis	Anti-GBM disease	COVID-19	Anti-GBM disease
**Treatment**	**PE (n)**	2				13		14
	**IAS (n)**	9						
	**IVS**	3 × 1000 mg				3 × 500 mg		
	**OS**	80 mg				80 mg		60 mg
	**IVCYC**	6 × 750 mg						1000 mg
	**R**					2 × 1 g		2 × 1 g
	**IVIG**			2 × 10 g	2 × 10 g			

*Abbreviations*: *PE* Plasma exchange, *IAS* Immunoadsorption, *IVS* Intravenous steroids, *OS* Oral steroids, *IVCYC* Intravenous cyclophosphamide, *R* Rituximab, *IVIG* Intravenous immunoglobulin

## Discussion and conclusions

Anti-GBM disease is very rare with reported incidence rates between < 1 to 1.79 per million population per year [1, 3, 4]. Although it is an autoimmune disease, reports of temporal and spatial clustering suggest that environmental factors such as infections may play a role in disease induction [4]. COVID-19 may be one such infection, as suggested by a report of a cluster of cases in London during the current pandemic. The authors report a five-fold increased incidence and four of eight patients had antibodies to SARS-CoV-2 [2]. Our case supports the assumption of a pathogenic link between COVID-19 and anti-GBM disease. The report from London and ours suggest that SARS-CoV-2 infection preceding anti-GBM disease is clinically mild or asymptomatic. There are, however, some unique aspects to our case. Whereas none of the cases in the London series had pulmonary involvement, our patient suffered from severe pulmonary haemorrhage. In contrast to the four reported London cases, our patient had a positive SARS-CoV-2 PCR test, but no antibodies against the virus were detectable. This is not unusual, as antibodies are detected with highly sensitive assays in about 90% of patients [5]. Negative tests are preferentially found in asymptomatic cases such as ours [6]. In addition, antibodies may go undetected during the first 2 weeks of infection [7].

At initial presentation the symptoms of respiratory distress, radiologic findings, the positive SARS-Cov-2 PCR and negative anti-GBM autoantibody ELISA suggested a diagnosis of COVID-19, and the patient was treated accordingly. Haemoptysis, however, is not a classical manifestation of COVID-19, which made us suspicious that the patient could instead have a recurrence of anti-GBM disease. This was eventually confirmed by renal histology. We therefore propose that the clinical symptoms and radiology findings were already caused by anti-GBM disease, and not COVID-19.

As SARS-CoV-2 PCR tests are 100% specific, there is no doubt that our patient had contracted the infection. The patient did not report any prodromal symptoms compatible with COVID-19 in the last few weeks. The onset of disease with pulmonary haemorrhage was rather abrupt. The positive SARS-CoV-2 PCR with a high cycle threshold suggests that the patient had contracted the infection only very recently, probably 2 to 3 weeks previous [8]. This means that anti-GBM disease occurred rather rapidly after the SARS-CoV-2 infection, probably because our patient was not having her first attack, but rather her second recurrence of anti-GBM disease.

SARS-CoV-2 infects pulmonary endothelial cells [9]. Consecutive complement activation and inflammation cause endothelial injury, leading to exposure of the basement membrane [10]. This sequence of events may release the NC1 antigen into the circulation. The autoantigen may then have stimulated long-lived memory plasma cells reacting to NC1 to produce and secrete autoantibodies, resulting in rapid anti-GBM disease recurrence in our patient. Proteinuria and haematuria have been described in a large proportion of patients with COVID-19, suggestive of renal, possibly glomerular damage during the infection [11]. Therefore, tissue damage in the alveoli in the lung and glomeruli in the kidney may make the autoantigens in the basement membrane accessible to circulating antibodies, eventually leading to pulmonary renal syndrome. Intercurrent infection could also lead to unspecific bystander activation of pre-existing autoreactive T and B lymphocytes [12]. In addition, any concurrent infection may precipitate or aggravate pulmonary haemorrhage and glomerular damage in anti-GBM disease [13]. A clear causal mechanism, however, between SARS-CoV-2 infection and anti-GBM disease has thus far not been established.

Rituximab is an effective alternative to cyclophosphamide in the treatment of anti-GBM disease [14, 15]. Rituximab was also successful in the treatment of the patient’s first relapse. She had already received cyclophosphamide for her initial attack. Therefore, we decided to change treatment from cyclophosphamide to rituximab in order not to increase the cumulative dose of this rather toxic medication in a young woman.

Another interesting aspect of our case is the continuous decrease of anti-GBM antibodies detected by antigen-specific ELISA during her initial disease and recurrences. At the first attack 11 years earlier, antibodies were detected at a high titre. During the first recurrence 5 years ago, autoantibodies were present, but only borderline. At the current flare-up, autoantibodies were undetectable. A kidney biopsy, however, clearly showed linear autoantibody binding to the glomerular basement membrane. We suggest that affinity maturation of the autoantibodies leads to their rapid and avid binding to the antigen and disappearance from blood, rendering them undetectable with conventional ELISA tests. Alternatively, chronic antigen stimulation may have caused a switch from IgG1 to IgG4 isotype anti-GBM antibodies, which are not detected by conventional ELISA [16, 17]. Further IgG subtype classification by immunochemistry in the renal biopsy would allow to identify these rare IgG4 antibodies but was not available.

Despite a negative autoantibody ELISA we decided to treat the patient with plasma exchange. Monitoring the efficacy and frequency of plasma exchange by antibody titres is not possible in that situation. The early relapse after seven plasma exchanges may be due to the strong and probably prolonged binding of the autoantibodies to the basement membranes.

In conclusion, we report a patient with anti-GBM disease, who had a recurrence of the disease after infection with the new coronavirus SARS-CoV-2, which confirms earlier reports that COVID-19 may be a trigger of this life-threatening autoimmune disease. More clinical and experimental investigations are necessary to further establish and confirm a causal link between these diseases. Initial differentiation between COVID-19 and anti-GBM disease may be challenging.

## Data Availability

Data sharing is not applicable.

## Abbreviations

SARS-CoV-2: Severe acute respiratory syndrome coronavirus 2
COVID-19: Coronavirus disease 2019
GBM: Glomerular basement membrane
ELISA: Enzyme-linked immunoassay
RT-PCR: Reverse transcription polymerase chain reaction
